# Effects of fish movement assumptions on the design of a marine protected area to protect an overfished stock

**DOI:** 10.1371/journal.pone.0186309

**Published:** 2017-10-12

**Authors:** Jorge Cornejo-Donoso, Baldvin Einarsson, Bjorn Birnir, Steven D. Gaines

**Affiliations:** 1 Interdepartmental Graduate Program in Marine Science, Marine Science Institute, University of California Santa Barbara, Santa Barbara, CA, United States of America; 2 National Center for Ecological Analysis and Synthesis, University of California Santa Barbara, Santa Barbara, CA, United States of America; 3 Department of Mathematics, South Hall, University of California Santa Barbara, Santa Barbara, CA, United States of America; 4 School of Engineering and Natural Sciences, University of Iceland, Reykjavik, Iceland; 5 Bren School of Environmental Science & Management, Bren Hall, University of California Santa Barbara, Santa Barbara, CA, United States of America; Bangor University, UNITED KINGDOM

## Abstract

Marine Protected Areas (MPA) are important management tools shown to protect marine organisms, restore biomass, and increase fisheries yields. While MPAs have been successful in meeting these goals for many relatively sedentary species, highly mobile organisms may get few benefits from this type of spatial protection due to their frequent movement outside the protected area. The use of a large MPA can compensate for extensive movement, but testing this empirically is challenging, as it requires both large areas and sufficient time series to draw conclusions. To overcome this limitation, MPA models have been used to identify designs and predict potential outcomes, but these simulations are highly sensitive to the assumptions describing the organism’s movements. Due to recent improvements in computational simulations, it is now possible to include very complex movement assumptions in MPA models (e.g. Individual Based Model). These have renewed interest in MPA simulations, which implicitly assume that increasing the detail in fish movement overcomes the sensitivity to the movement assumptions. Nevertheless, a systematic comparison of the designs and outcomes obtained under different movement assumptions has not been done. In this paper, we use an individual based model, interconnected to population and fishing fleet models, to explore the value of increasing the detail of the movement assumptions using four scenarios of increasing behavioral complexity: a) random, diffusive movement, b) aggregations, c) aggregations that respond to environmental forcing (e.g. sea surface temperature), and d) aggregations that respond to environmental forcing and are transported by currents. We then compare these models to determine how the assumptions affect MPA design, and therefore the effective protection of the stocks. Our results show that the optimal MPA size to maximize fisheries benefits increases as movement complexity increases from ~10% for the diffusive assumption to ~30% when full environment forcing was used. We also found that in cases of limited understanding of the movement dynamics of a species, simplified assumptions can be used to provide a guide for the minimum MPA size needed to effectively protect the stock. However, using oversimplified assumptions can produce suboptimal designs and lead to a density underestimation of *ca*. 30%; therefore, the main value of detailed movement dynamics is to provide more reliable MPA design and predicted outcomes. Large MPAs can be effective in recovering overfished stocks, protect pelagic fish and provide significant increases in fisheries yields. Our models provide a means to empirically test this spatial management tool, which theoretical evidence consistently suggests as an effective alternative to managing highly mobile pelagic stocks.

## Introduction

Marine Protected Areas (MPAs) are a spatial management tool commonly used to restore and protect populations of marine organisms. When scaled to the proper size or combined as an interconnected network, they can effectively protect fisheries stocks and increase fisheries yields [1–3]. Yet, to be successful for both conservation and fisheries goals, MPA designs must adequately address the consequences of species movement, including swimming behavior of adults and dispersal of larvae [4,5].

Theoretical studies suggest that a well-designed MPA can provide comparable benefits to those obtained with perfect management of the catch or they can even exceeded under the right conditions [3]. For example, Hasting and Botsford [6] showed that for species with sedentary adults and larval dispersal, the optimal MPA size can offer identical stock protection and yields to those provided by the optimal fishing mortality rate. Several other authors have corroborated this result, concluding that most of the fisheries benefits of MPAs are obtained when adults have medium to low annual movement, and these benefits are reduced as the movement rate increases [1,2,7]. Nevertheless, Gaines *et al*. [4] indicates that one of the main requirements for an effective MPA is an area size proportional to the movement rate of the organisms, suggesting that MPAs can be effective in protecting highly mobile organisms if designed with larger areas that exclude extractive activities.

Although it is generally accepted that MPAs can provide multiple benefits if well designed, it is a challenge to link any gains specifically to the MPA because it requires a long time series of inside/outside and before/after data [8]. As a result, empirical evaluations of MPAs are uncommon. To overcome this limitation, simulation approaches have been proposed as a complementary way to compare potential MPA designs, but they are not exempt from challenges. For example, simulations are highly sensitive to the assumptions describing the biology of larval dispersal, juvenile/adult and fisheries movement [2,3,5,7,9,10], and to the complexity of the natural environment and of human driven systems [11]. Furthermore, most MPA simulations have historically been done for benthic organisms with slow or sessile adults, where the assumption of simple diffusive or advective movement is a rational approach to depict passive dispersal of particles like eggs or larvae [12–16].

Thanks to the advances on computer modelling, simulation approaches with complex and detailed movement dynamics are now possible [12,17]. This capability to describe detailed movement dynamics, including those at the individual level, allows the implementation of realistic MPA simulations and the prediction of their effectiveness in stock protection and yields increase [18–20].

There is an increasing number of publications that report simulations of individual movements dynamics on a heterogeneous space. In these studies, the main assumption is that each individual fish imitate the movement of neighboring fishes, forming aggregations that then respond to environmental forcing (e.g. temperature, salinity or food gradients) [21]. As individual based models (IBM) allow the simulation of fishery dynamics at a very fine spatial and temporal scale [22], it is possible, for example, to recreate complex movement dynamics including migration [19,23,24], predict spatial distribution from low to highly mobile organisms [20,25] and test competing MPA designs [19].

It has been argued that predictions from MPA simulations can be sensitive to simplifying movement assumptions [5,9,10], but it is expected that this problem will be minimized with the inclusion of detailed movement dynamics in MPA simulations. However, even as the number of publications about MPA simulations using IBM increases, a systematic evaluation of the benefits of including such movement complexity, especially as compared to the predicted outcomes of an MPA model using simplified movement assumptions (i.e. assuming diffusive movement), has not yet been made.

In this study, we developed an IBM that explicitly accounts for larvae and juvenile/adult movement, spatial-temporal fishing dynamics and for individual fish age-dependent natural mortality, including fecundity and density dependent survival from eggs to yearlings. The main goal of these simulations is to identify the benefits of using complex movement dynamics instead of simplifying assumptions, and explore the potential of large MPAs to protect stocks and increase yields of a pelagic organism.

## Material and methods

### Model outline

The IBM used in the present work is an adaptation of the particle interaction model introduced by Vicsek and colleagues [26], extended by Czirók and Vicsek [27], and modified by Magnússon [21]. The dynamical system analysis of the corresponding ordinary differential equations model was done by Birnir [28].

The model was used to explore the effects of alternative movement assumptions (for a pelagic organism) on the optimal size of an MPA where no extractive activities are allowed. For this goal, we developed a complex movement model where each individual fish adjusted their movement based on the direction and speed of neighboring fishes, allowing the formation of schools that then adjust their movement to respond to a spatially heterogeneous environmental forcing. The model also included population dynamics and the effects of spatially dynamic fishing on population mortality. The fishing ground was represented by a 2D space discretized in 100 by 40 sectors. We defined four comparable models with movement assumptions (scenarios) of increasing complexity:

1. *Random movement of adults and eggs/larvae*: this scenario is equivalent to movement by diffusion, with a mean diffusion coefficient of 0.0069 sectors per day. Eggs, juveniles and adults have the same pattern of movement but differed in their swimming speed.
2. *Aggregation*: in this scenario adults interact with their neighbors, coordinating their speed and direction, forming aggregations. The direction and speed of the school is not influenced by any environmental forcing, nor transported by currents.
3. *Aggregation and environmental forcing*: in this case adults interact with their neighbors, forming aggregations that coordinate their swimming direction and speed. These aggregations react to environmental forcing (SST) by adjusting their direction and speed.
4. *Aggregation*, *environmental forcing*, *and transport by currents*: in this case, adults interact with their neighbors, forming aggregations that coordinate their swimming direction and speed. These aggregations react to environmental forcing (SST) by adjusting their direction and speed and are transported by currents. Eggs/larvae are also transported by the currents, but do not form aggregations.

In all scenarios, the state variables for each fish are position, speed, movement direction, and age. In the most complex model, fish sense the position of nearby fishes and the local temperature gradient. Then, based on these stimuli, the movement direction and speed is actively adjusted for the next time step, while currents passively transport them.

At the population level, the state variables are the intrinsic growth rate, carrying capacity, natural mortality rate (*Z*), and fishing mortality rate (*F*; Table 1).

**Table 1:** Model input values.

Parameter	Initial Value	Units
Initial		
Number of Fish	*~25*,*000*	*ind*.
Reproduction Day	*N*(250, 20)	*Day*
Fish Heading	*U*(0, 360)	*Deg*
Adult Fish Speed	*U*(0.42, 0.50)	*sector d* ^*-1*^
Eggs/Larvae speed	*U(0*, *0*.*1)*	*sector d* ^*-1*^
Initial fish age	*U*(1, 3)	*year*
Diffusion Coefficient	0.0069 (0.0000, 0.0167)	*sector d* ^*-1*^
Natural mortality (*Z*)		
1 to 4 years old	7x10^-4^	*d*^*-1*^
5+ years old	3x10^-3^	*d*^*-1*^
Fishing mortality (*F*)	1.27	*y*^*-1*^
Fishing activity start	20	*year*
Management activity start	30	*year*
Total simulation length	70	*year*
Fecundity	10	*eggs ind* ^*-1*^
Radius of		
Repulsion *r*_*r*_	0.02	*sector*
Alignment *r*_*o*_	0.10	*sector*
Attraction *r*_*a*_	0.10	*sector*
Temperature preferences *[T*_*1*_, *T*_*2*_*]*	(16, 18)	*°C*
Weighted influence of		
Neighbors (α)	0.995	
Temperatures (β)	0.005	
Boat aggregation index (c)	3	
Fish carrying capacity (*K*)	30,000	*ind*.
Intrinsic rate of increase (*r*)	1.2	*ind*. *year* ^*-1*^
Simulation time-step *Δt*	0.2	*day*

The model incorporates stochasticity in several components including: the initial direction and movement speed when a new fish recruits to the system (age 0), the timing of individual death and reproduction. Natural and fishing mortality rates are implemented as a daily probability of death for each fish; therefore, at any given time *t*, the natural and fishing mortality of an individual fish is unknown, but the daily mean of these two variables is predefined and known.

### Population dynamics

The fish demographic parameters used in the simulations are based on the Peru-Chile anchovy (*Engrulis ringens* Jenyns, 1942). This small pelagic fish has a common length of ~15 cm, a short, iteroparous life cycle, and a main spawning event occurring around the second half of the year (Table 1). Our theoretical fish was based on anchovy, because they represent one of the most important fisheries in the world, have been subject to intense fishing for decades, have a short life cycle and fast reproduction [29], which are all characteristics that make it an ideal study case for the efficacy of a pelagic MPA to protect and manage the stock.

Adult natural mortality is defined for two age class ranges: one to four years old and five years and older (Table 1). A total of three fish stage classes are included in the model: eggs/larvae, juveniles and adults. Each stage class is defined by distinct swimming capacities, aggregation behaviors, and responses to environmental forcing.

1. *Eggs/Larvae* are not active swimmers; therefore, they cannot form aggregations or respond to temperature fields. Their movements in the system are a consequence of transport by currents and an initial slow movement that spreads them from the spawning point. These organisms are between one day and six months old.
2. *Juveniles* have limited swimming capacity; they form aggregations and respond to temperature fields. Their maximum speed is slower than adults. Juveniles are not fished (*F* = 0). These are organisms between six months to one year old.
3. *Adults* swim faster than juveniles. They are recruited to the fishery (*F* > 0) and reproduce once a year. These organisms are 1 year and older.

The model simulation starts on January 1^st^, with a time step of $\frac{1}{5}$ of a day. Reproduction is defined as a population event normally distributed around September 7 (day 250) and a standard deviation of 20 days (Table 1) [30–32]. As the fish is added to the system, a reproduction day is assigned such that reproduction will occur not before the fish reaches one year (age of maturity), with a defined fecundity of 10 eggs per individual. After the reproduction event, eggs are subject to density dependent natural mortality which is adjusted to satisfy the dynamics as predicted by a Gordon Schaefer model [33] and calculated as the difference between the expected number of fish for next year (*N*_*t+1*_ *= Rec*_*t+1*_*+N*_*t*_), the effective number of recruited fish (adults) at year t (*N*_*t*_) and the number of eggs/larvae ($N_{eggs_{d}}$) in the system on day *d*. In this way, the Gordon Schaefer model allows a simple probabilistic approach to estimate the daily survival probability of an individual egg/larva which is obtained as:

$$Rec_{t+1}={r\times N}_{t}\left(1-\frac{N_{t}}{K}\right)$$ (1)

And therefore, the daily probability of survival (*S*_*prob*_) is:

$$S_{prob}=\frac{Rec}{N_{eggs_{d}}}$$ (2)

**Fig 1:**
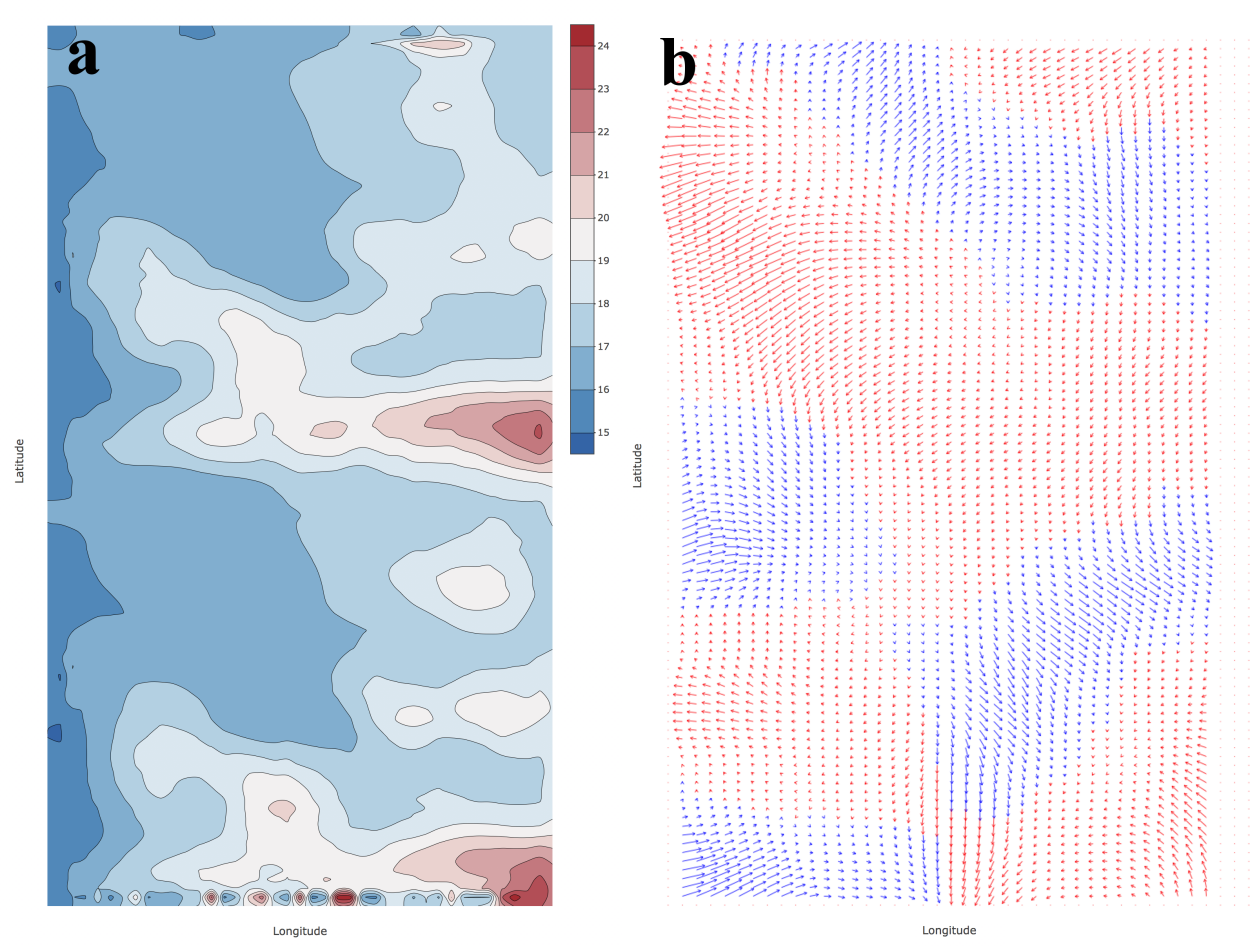
Maps of the environmental forcing used for the simulation scenarios that included a) sea surface temperature (in °C) and b) surface currents (red westward and blue eastward).

The population is defined to move in a two-dimensional field (no vertical movement included) in a rectangular grid of 100 by 40 sectors that represented the ocean. This simulated ocean is designed to cover a geographic area larger than the expected distribution range of the stock so it functions as a closed system, with no immigration or emigration (i.e. repelling borders). A monthly mean sea surface temperature (*SST*) for August of 2009 was obtained from the global dataset provided by NASA (OceanColor web site; Daytime image *SST* 11μ 4x4 km processed from the data obtained with the MODIS-Aqua sensor, https://oceancolor.gsfc.nasa.gov/) and used as the movement environmental forcing (Fig 1A). This month had significant spatial *SST* variability with values above and below the range used to force the movement (Eqs 9 and 11), resulting in a spatially heterogeneous environment with fixed temporal variability. A subset of the global *SST* data was selected off the coast of Peru and Chile that covers the Peru-Chile anchovy stock distribution area, approximately between the 18° and 24° Lat S. A mask was applied to remove and reshape the land and ocean areas, making them equivalent to the defined simulation area.

Ocean currents were modelled as the annual mean of AVISO/geostrophic currents (NOAA Coastwatch; https://coastwatch.pfeg.noaa.gov/coastwatch/CWBrowserWW360.jsp) for the same region of the Peruvian and Chilean coasts. These data have a resolution of 0.25° and were interpolated and reshaped to make them compatible with the simulation area (using kriging in R [34]; Fig 1B).

### Simulation description

**Fig 2:**
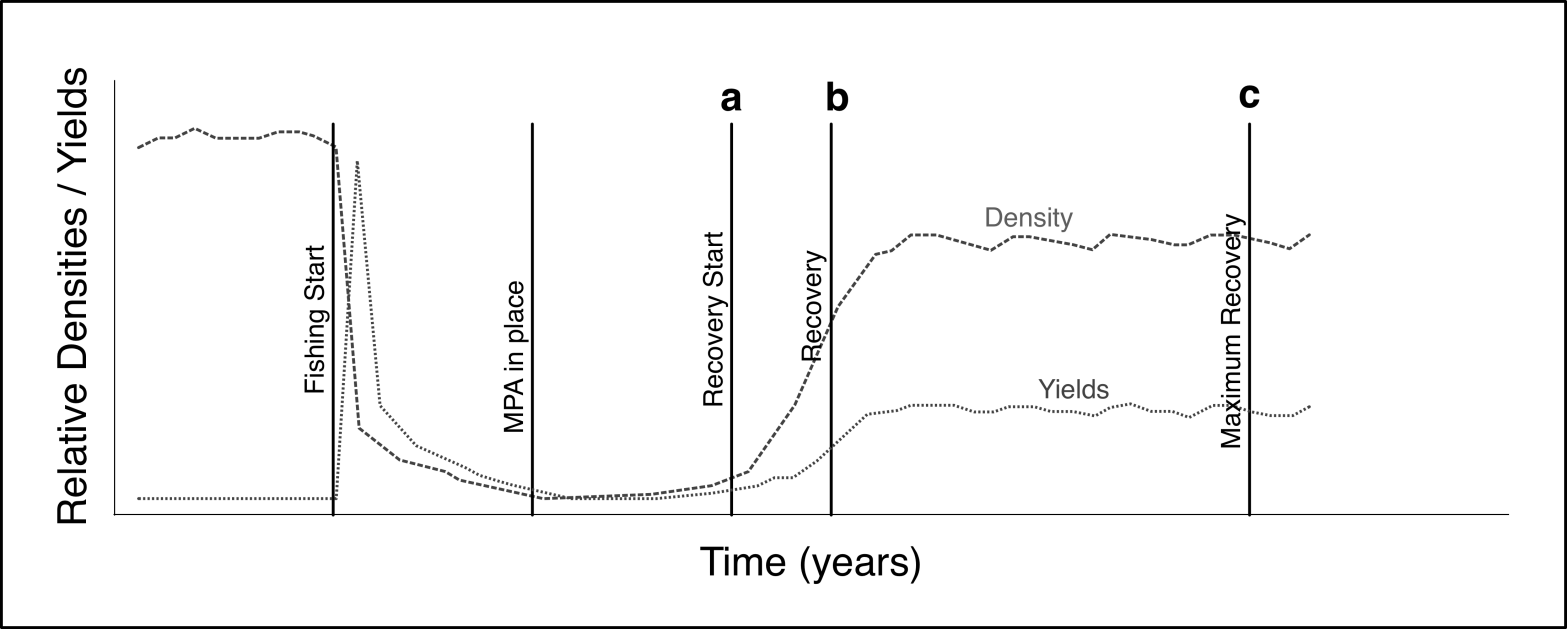
Representation of a relative stock density (dashed line) and fisheries yields (dotted line) time series. The figure shows when: the fishing start (year 10), the MPA is implemented (year 20), the recovery start (year 35, a), the density and yield is increasing fast (year 40, b), and the recovery is at its maximum and has reached the equilibrium (year 65, c).

All simulations run for 70 years and are started by assigning a random position, speed, direction, day of future reproduction, and age to each fish in the system (Table 1). During the first 20 years fishing mortality was set to zero to allow simulations to stabilize and remove potential effects of the initial conditions. At year 21, the entire area is subject to fishing and continued until the year 31, when an MPA is implemented as an east-west strip at the center of the simulated region (Fig 2). Simulations were run with an increasing fraction of the total area closed as an MPA to find the optimal size in terms of fisheries yields. A fixed fraction of the area is therefore excluded from fishing, while the fishing fleet is allowed to operate all year around outside the MPA.

For each time step, fish interact with their neighbors and respond to the temperature field by adjusting their heading and speed. As the fish swim, they are also transported by currents. At the beginning of every day, fish age by one day, natural mortality and reproduction occur within the entire fish population, and then the fishing fleet is redistributed and imposes fishing mortality.

When a fish reproduces, the new eggs/larvae are given a random heading and movement speed at birth (Table 1). Eggs and larvae are only transported by currents, while juveniles also swim with limited abilities during their first six months. If the juvenile survive the development period, the fish’s swimming capacity progressively increases until reaching maturity (one year old), when they are recruited to the fishery (*Engraulis* spp) [29]. At this point, fish are susceptible to fishing mortality based upon the daily patterns of fish and fleet spatial distribution. If a fish survives to its designated reproduction date, it releases offspring to complete the cycle.

### Spatial distribution of fishing effort

The fishing fleet movement was modelled as in Hilborn *et al*. [7]. Under the assumption that fishermen concentrate their effort where highest captures are expected, fishing effort was allocated in the grid as proportional to adult fish abundance:

$$B_{ijt}^{\prime }=\left\{ \begin{array}{ll} exp\;\left[-c(1-\frac{N_{ijt}}{M_{Nt}})\right] & if\;ij\;is\;outside\;the\;MPA\\ 0 & if\;ij\;is\;inside\;the\;MPA \end{array}\right.$$ (3)

*c*: Aggregation parameter for effort, between 1 and 5. Determines how effort respond to fish abundance, high values result in higher concentration of effort in density hotspots
*M*_*Nt*_: Maximum abundance of adult fish in the grid outside the MPA at the time *t*
*N*_*ijt*_: Number of adult fish in sector *ij* at time *t* before the redistribution takes place

Using the spatial distribution of effort, 5,000 fishing boats are redistributed in the space as:

$$B_{ijt}=5,000\frac{B_{ijt}^{\prime }}{\sum_{ij}B_{ijt}^{\prime }}$$ (4)

Where:

*B’*_*ijt*_: Relative effort in sector *ij* at time *t*
*B*_*ijt*_: Number of fishing boats in sector *ij* at time *t*

Eq 4 explains how fishing boats concentrate in response to fish densities in a particular sector. Parameter *c* (Eq 3) was set to three for all simulations, which represents an intermediate level of fishing boat aggregation in areas of high fish densities [7]. For each sector in the simulation area, the spatially explicit fishing effort is obtained by multiplying the daily fishing mortality rate by the number of boats in a particular sector and by a scaling factor that characterizes the likelihood of capture for a given density of fish and a given effort of fishing calculated as in White and Costello [35]. As a result, an individual fishing mortality probability is obtained, which is homogeneous for any *ij* pixel at time *t*, but heterogeneous between them.

The annual fishing mortality for our theoretical fishery was set an average of 1.27 per year over the space and time, equivalent to the fishing mortality reported for the Peru-Chile anchovy fishery [36–38]. This high fishing mortality is appropriate to drive the stock to collapse and provides the conditions to test the benefits of the MPA to induce recovery and protect the stock.

### Fish movement

**Fig 3:**
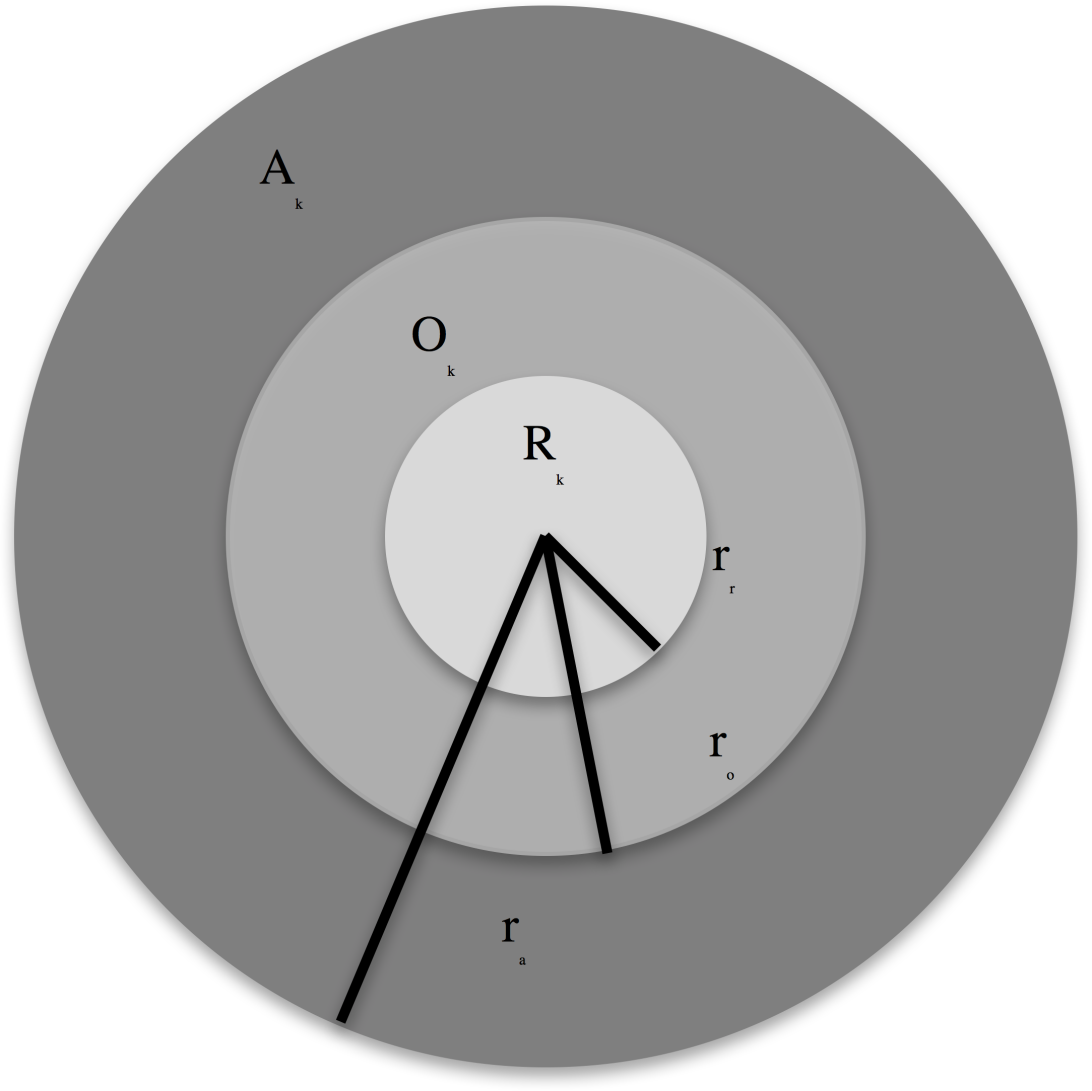
Zones of interaction of particle *k*. *A*_*k*_ is the *zone of attraction* (dark grey area), *O*_*k*_ is the zone of orientation (medium grey) and *R*_*k*_ if the *zone of repulsion* (light grey area). Each zone has a radius of *r*_*a*_, *r*_*o*_ and *r*_*r*_ respectively.

The interactions among fish are simulated using three sensory zones around the fish (Fig 3)[23,39,40]. The innermost region is the *zone of repulsion* (*R*_*k*_, light grey). In this region fishes head away from each other, avoiding collisions. The intermediate region is the *zone of orientation* (*O*_*k*_, medium grey) where fishes align in speed and direction. Finally, the outer region the *zone of attraction* (*A*_*k*_, dark grey) where fishes head toward each other, forming aggregations.

The number of fish in the repulsion, orientation and attraction zones is denoted by $N_{R_{k}}$, $N_{O_{k}}$ and $N_{A_{k}}$, respectively; ***q***_*k*_(*t*) *=* (*x*_*k*_(*t*),*y*_*k*_(*t*))^*T*^ is the vector of fish positions, *v*_*k*_(*t*) is its speed, and *ϕ*_*k*_ is the direction of fish *k* at time *t*.

A fish *k* updates its speed by:

$$v_{k}(t+∆t)=\frac{1}{N_{O_{k}}}\sum_{j\in N_{O_{k}}}v_{j}(t)$$ (5)

and its position is updated by:

$$q_{k}(t+∆t)=q_{k}(t)+∆t\cdot v_{k}(t+∆t)\left(\begin{array}{c} cos(\phi_{k}(t+∆t))\\ sin(\phi_{k}(t+∆t)) \end{array}\right)$$ (6)

For each time step (*Δt*), the heading and direction of the fish is recalculated based on the previous position and the positions of neighboring fish. Movement occurs in continuous space and is initialized as in [41,42]. A weighted average of the direction and speed is taken over the zone of orientation and *ϕ*_*k*_ (*t+Δt*) is calculated as

$$\left(\begin{array}{c} cos(\phi_{k}(t+∆t))\\ sin(\phi_{k}(t+∆t)) \end{array}\right)=\frac{d_{k}(t+∆t)}{\left\|d_{k}(t+∆t)\right\|}$$ (7)

where:

$$d_{k}(t+∆t)≔\frac{1}{N_{R_{k}}+N_{O_{k}}+N_{A_{k}}}\times \left(\sum_{r\epsilon N_{R_{k}}}\frac{q_{k}(t)-q_{r}(t)}{\left|\left|q_{k}(t)-q_{r}(t)\right|\right|}+\sum_{o\epsilon N_{O_{k}}}\left(\begin{array}{c} cos(\phi_{O}(t))\\ sin(\phi_{O}(t)) \end{array}\right)+\sum_{a\epsilon N_{A_{k}}}\frac{q_{a}(t)-q_{k}(t)}{\left|\left|q_{a}(t)-q_{k}(t)\right|\right|}\right)$$ (8)

‖…‖ represent the vector norm in two dimensions (e.g. $\|d\|=\surd \sqrt{d_{1}^{2}+d_{2}^{2}}$), *q*_*r*,_ *q*_*a*_ are the fish position in the areas of repulsion and attraction (Eq 6) and *ϕ*_*k*_ is speed and direction of those in the zone of orientation (Eq 7).

The environment is incorporated as a grid of currents and temperature data. Currents move the fish directly, and this translation movement is independent of the fish’s own movements in response to other fish and to temperature. The current field is denoted by ***C***(*i*,*j*), where *i* and *j* are the coordinates where that particular current value is found. Fish responds to the temperature field ***T***(*i*,*j*) by seeking to find locations within their preferred temperature range, *T*_*1*_ to *T*_*2*_. The fish adjust to the surrounding temperatures according to the gradient of the function *r*

$$r(T)≔\left\{ \begin{array}{ccc} -{\left(T-T_{1}\right)}^{2} & if & T\leq T_{1}\\ 0 & if & T_{1}\leq T\leq T_{2}\\ -{\left(T-T_{2}\right)}^{2} & if & T_{2}\leq T \end{array}\right.$$ (9)

Including the effects of the environmental fields on the particle’s positions (***q***_*k*_(*t*)) we obtain

$$q_{k}(t+∆t)=q_{k}(t)+∆t\cdot v_{k}(t+∆t)\frac{D_{k}(t+∆t)}{\left|\left|D_{k}(t+∆t)\right|\right|}+C(q_{k}(t))$$ (10)

where:

$$D_{k}(t+∆t)≔\alpha \frac{d_{k}(t+∆t)}{\left|\left|d_{k}(t+∆t)\right|\right|}+\beta \frac{\nabla r(T(q_{k}(t)))}{\left||\nabla r(T(q_{k}(t)))|\right|}$$ (11)

*∇* represent the two-dimensional gradient of the vector ***r*** (e.g. *∇r* = ∂_*x*_*r* + ∂_*y*_*r*) and the speed (*v*_*k*_) is calculated as in Eq 5, ***d***_*k*_ is the same unit vector as in Eq 7, and the weights satisfy the following

α+β=1 (12)

where, *α* corresponds to the particles’ interactions with their neighbors and β defines the responses to the temperature field.

This grid includes the border as areas of extreme temperature values, which repel the fish and contain them within the grid.

The simulation started with *ca*. 25,000 randomly distributed fish. Because of stochasticity in several elements of the simulation (i.e. movement heading and speed, day of reproduction, natural and fishing mortality), eleven replicates were run to explore this variability.

### Sensitivity analysis

A sensitivity analysis was performed for the three most important parameters used in the simulations: boat aggregation, female reproduction potential, and fishing mortality rate. For this purpose, the scenario which includes aggregations, responses to the environment, and transport by currents was used under the assumption that it would be the most sensitive to parameter changes.

The sensitivity analysis was performed by modifying one parameter at a time, using an MPA of 30% and comparing the results for density and yields (average of 10 runs) after 15, 25 and 45 years of the MPA implementation.

The values used for each parameter were:

1. Boat aggregation *c*: By definition (Eq 3) bounded to integers values between one and five [7], each of them tested.
2. Female reproduction potential: This is a positive integer that represent the number of eggs produced by a female. It affects the individual natural mortality rate as consequence of density-dependent survival of pre-recruits. For the sensitivity analysis, the range between five and 15 eggs was tested.
3. Fishing mortality rate *F*: The fishing mortality rate was applied to all the adult fish in the system. The values used in this analysis were 1.10, 1.20, 1.27, 1.30, 1.35, 1.40 and 1.50 year^-1^.

The simulation model was developed using C++ and the source code is available at GitHub (https://github.com/cornejotux/MPA-and-fish-movement).

## Results

**Fig 4:**
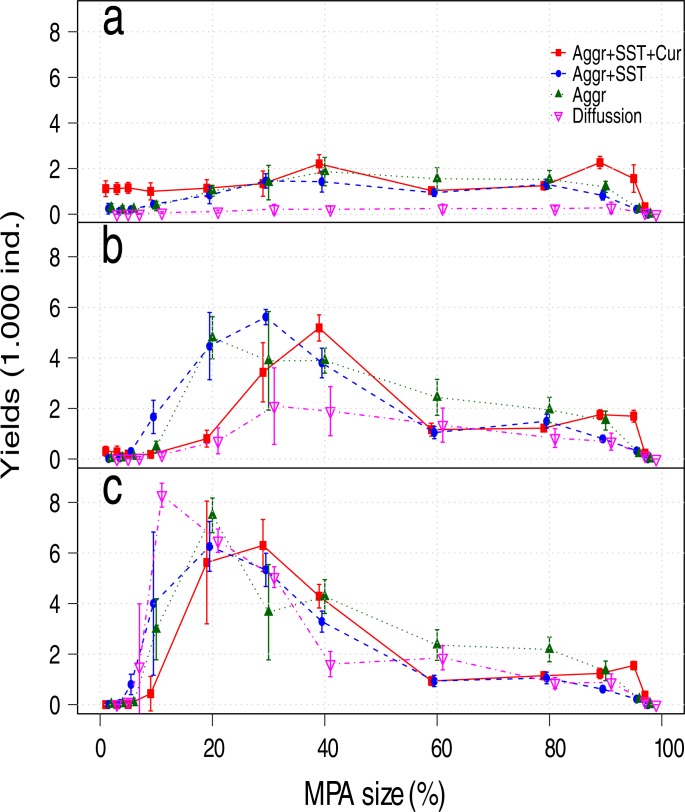
Mean landings values by MPA size and scenarios after a) 15, b) 20, and c) 45 years of MPA implementation. Aggr: Aggregation behavior, SST: Responses to the sea surface temperature, and Cur: Transport by currents.

**Fig 5:**
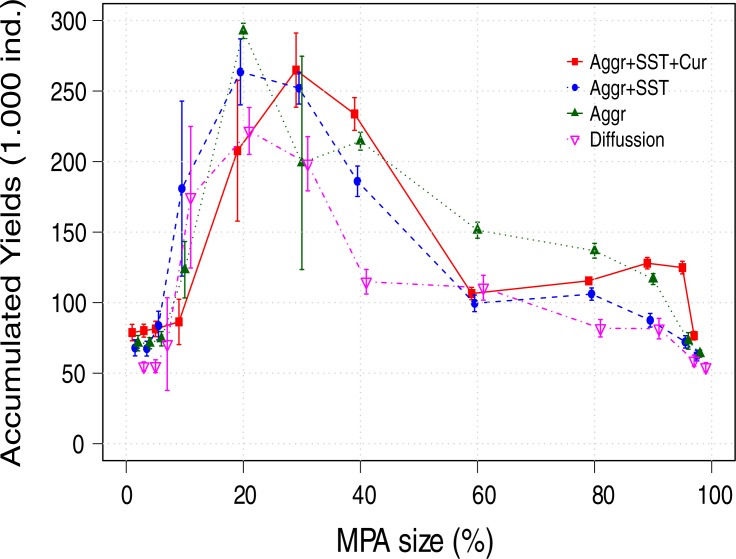
Accumulated landings for the whole period after MPA implementation (50 years). Aggr: Aggregation behavior, SST: Responses to the sea surface temperature, and Cur: Transport by currents.

**Fig 6:**
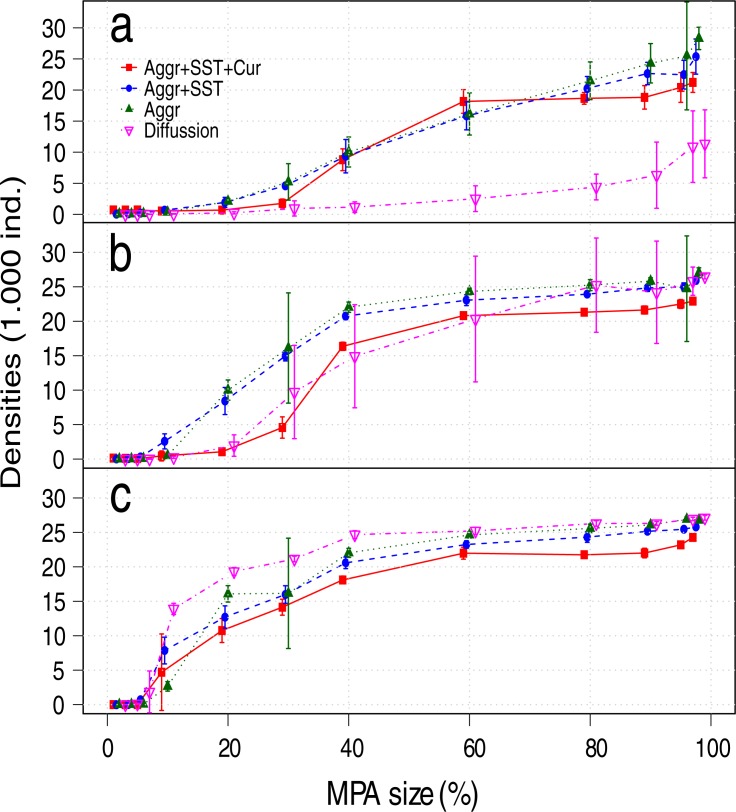
Mean densities by MPA sizes and scenario after a) 15, b) 20, and c) 45 years of the implemented MPA. Aggr: Aggregation behavior, SST: Responses to the sea surface temperature, and Cur: Transport by currents.

The differences in MPA size that maximize long term fishery yields between scenarios were small, with a range between 10% and 30% (Fig 4), or 20% to 30% when accumulated yields are considered (Fig 5). MPAs areas smaller than 10% had little or no benefits under any movement assumption (Figs 4, 5 and 6).

Although the differences in the optimal MPA size that maximize the fishery yields were modest across movement scenarios at equilibrium (Fig 4C), the trend was that larger MPAs were required as movement complexity increased; the maximum fisheries yields were obtained with an MPA of 10% for the diffusion scenario (~90% of maximum sustainable yield; $MSY=\frac{K\cdot r}{4}$, where *K* is the carrying capacity and *r* the intrinsic growth rate), 20% for aggregations and for aggregations forced by *SST* (~80% of MSY), and 30% when fish aggregations forced by *SST* and transported by currents were assumed (~80% of MSY). The opposite trend was observed for the mean stock recovery time, where the diffusive movement scenario presented the slowest recovery (Figs 4B and 6B). These differences observed in the mean recovery time between scenarios can result in underestimations in yields as large as 30% for the optimal MPA size (Fig 5) when the accumulated fisheries benefits are considered.

The increase in the fishery yields, their peak and later decrease (Figs 4C and 5) is evidence that under optimum size, large MPAs can be effective as management tools to protect highly mobile stocks (e.g. pelagic), increasing their biomass and delivering benefits to the fishery. Our results also show that when 60 to 80% of the area was protected, the reduction of fishing grounds was compensated by a larger fish stock and the consequent spill-over effect, resulting in relatively constant fishing yields (Fig 4). Additionally, even when the protection of areas larger than those that maximize fisheries yields was suboptimal, they provided other benefits like a reduction in density and yields variability, and constant yields over time (Table 2).

**Table 2:** Summary of density (Dens) and yield estimations per Scenario and MPA size, with their respective standard deviation (SD). Int: Interaction between organisms, SST: Responses to the sea surface temperature, and Cur: Transport by currents.

MPA(%)	Scenarios															
	Diffusion				Interaction				Int+SST				Int+SST+Cur			
	Dens±SD		Yield±SD		Dens±SD		Yield±SD		Dens±SD		Yield±SD		Dens±SD		Yield±±SD	
2	11	±9	16	±14	8	±7	11	±9	7	±5	9	±8	4	±4	2	±1
4	124	±87	114	±74	29	±12	35	±13	43	±26	44	±33	5	±3	4	±4
6	1.854	±3.032	1.508	±2.481	72	±23	77	±23	710	±367	800	±404	8	±5	7	±5
10	13.898	±814	8.288	±472	2.650	±690	2.982	±1.209	7.851	±1.937	3.997	±2.832	4.706	±5.550	435	±683
20	19.330	±568	6.487	±462	16.076	±1.190	7.487	±687	12.715	±1.621	6.262	±988	10.757	±1.738	5.625	±2.428
30	21.144	±409	5.045	±412	16.153	±8.004	3.656	±1.886	15.981	±1.272	5.333	±656	14.144	±1.159	6.297	±1.029
40	24.674	±571 515	1.604	±499	22.028	±655	4.275	±669	20.578	±839	3.286	±418	18.096	±558	4.292	±467
60	25.220	±515	1.884	±478	24.624	±442	2.358	±605	23.243	±548	938	±223	21.979	±869	936	±180
80	26.294	±401	855	±218	25.584	±619	2.183	±489	24.313	±755	1.056	±230	21.748	±557	1.147	±162
90	26.290	±268	881	±335	26.077	±373	1.336	±389	25.153	±327	611	±127	22.003	±697	1.234	±206
96	26.947	±251	126	±25	26.906	±358	210	±76	25.445	±380	222	±61	23.191	±409	1.547	±168
98	27.120	±335	0	±0	26.773	±229	0	±0	25.818	±218	2	±1	24.265	±410	375	±157

**Fig 7:**
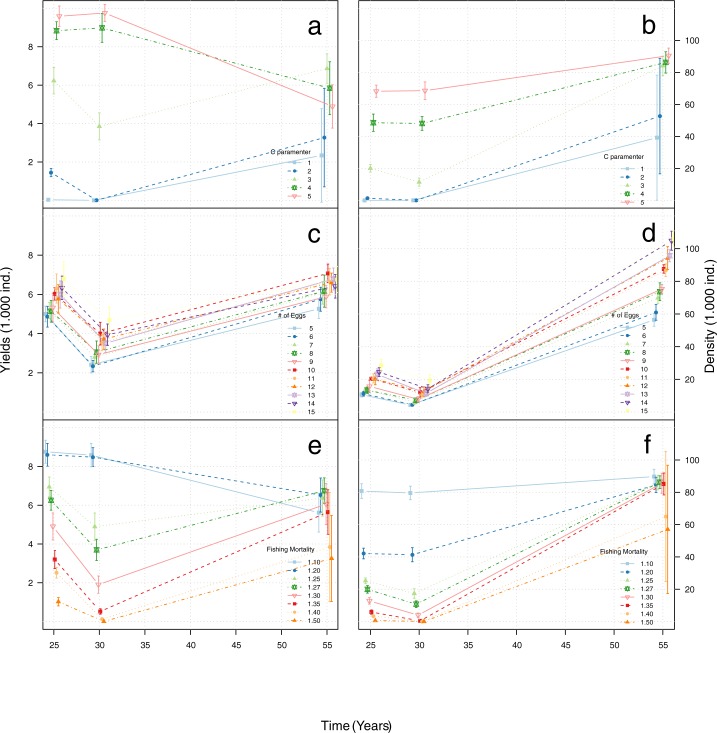
Sensitivity analyses using yields (a, c and d) and densities (b, d and f) for the sensitivity analysis of the boat aggregation parameter *c* (a and b), female reproduction potential *# eggs* (c and d) and fishing mortality rate *F* (e and f). Each line is an average of 10 runs with the input parameter as shown in the legend, vertical bars represent the standard deviation of the mean, obtained with ten simulation runs.

The results of the sensitivity analysis show that the density and yields obtained with the different values of boat aggregation and fishing mortality were sensitive shortly after the MPA implementation (15 and 25 after MPA), converging to similar values towards the end of the simulated period (Fig 7A, 7B, 7E and 7F). This general pattern was not observed for the female reproduction potential. In this case, density and yields values were similar shortly after the MPA implementation, maintaining similar yield values towards the end of the simulated period (Fig 7C), while the differences in fish densities increased as the time passed (Fig 7D).

## Discussion

The complexity involved in the movement of organisms within marine environments makes their spatial management challenging, and this has sparked a fruitful debate about the viability and effectiveness of such approaches [43]. Spatial management, and in particular marine protected areas, has been predominantly used to protect coastal and benthic sessile or low mobility organisms [44], while pelagic stocks have been historically managed using traditional effort control. Only in recent years has there been a rise in the interest of using large MPAs to protect pelagic stocks [35,45–49], especially those targeted by industrialized fisheries.

In this paper, we developed a complex model that allows the use of a wide range of movement assumptions (i.e. from diffusion movement to complex schooling behavior forced by the environment) to study how movement assumptions used in MPA simulations affect their design and the expected outcomes from their implementation in terms of stock size, fisheries yield, and recovery times. The model provides a platform to compare perceived costs and benefits of an MPA designed using simplified assumptions versus MPA designs that include complex and realistic movement dynamics.

The assumption that the most complex scenario (i.e. aggregations, SST and currents) is the closest representation to the real movement dynamics, allowed us to define it as the benchmark to compare all the simulated scenarios; in this way our results indicate that simplified movement models (e.g. random walk, diffusion, complete redistribution) overestimate the expected protection and fisheries benefits, as well it underestimate the required MPA size to protect the stock, with consequent suboptimal results. It is generally accepted that any increase in the understanding of individual and stock movement can improve an MPA design, and therefore its effectiveness [19]. Nevertheless, by comparing between the benefits obtained by the MPA (in terms of stock protection and fisheries yields) when complex movement dynamics were included (i.e. equivalent to increased fish movement knowledge), this research illustrates that complete knowledge of the fish movement dynamics is not a requirement for a successful MPA design [46]. If it is not feasible to implement a complex model to represent in detail the movement dynamics of a species, due to data constraints or other limitations, a simplified movement assumption can still provide guidelines for selecting the minimum MPA size that would protect stocks and benefit fisheries, and typically this approach gives results close to one third of the area, which has been proposed previously as a rule of thumb [3,4]. For example, when optimizing the MPA size using the diffusion model, the stock is predicted to rebuild to ~1/3 of the unfished densities, while doubling the protection area, as suggested by the full environment scenario, the stock size is predicted to increase to ~1/2 of unfished densities with yields of ~80% of MSY (Table 2).

The MPA sizes described here were equivalent or smaller than previously reported as optimal for fisheries benefits [4], but these areas are not small in absolute terms. For instance, considering the distribution area described for the anchovy stock shared by Peru and Chile [50], an MPA of 20% would be of ~40.000 km^2^, which is equivalent to 40 times the total area of the Channel Islands Marine Protected Areas network in California, or *ca*. 10% the size of the U.S. Marine National Monument Papahãnuamokuãkea. However, although the design and implementation of such large MPAs present significant challenges, their use as a fishery management tool have the advantage, with respect to the traditional management, to buffer against errors in stock assessment [51] and are considerably less data intensive when in place. Annual stock assessments and minimum size quotas are economically expensive, time consuming, and subject to high natural variability and uncertainty, particularly for small-pelagic fisheries with periodic regime shifts. Errors in stock assessment and quotas can be less relevant for the sustainable management of the stock if the MPA is properly designed.

Our results provide evidence for the potential success of large MPAs to protect and manage pelagic stocks, and of a direct relationship between marine reserve size, scales of animal movements, and reserve effectiveness [4,19], which has never been empirically tested before [19]. By removing some of the limitations recognized to MPAs fields studies (e.g. few data points, limited number of species and limited control of fishing mortality outside the MPA)[52], our results allow a comparison between MPA sizes and movement assumptions, identifying some of the conditions that can make them effective, and highlighting the relevance of empirical observations to corroborate the theory behind large MPAs. Even when the results of the sensitivity analysis suggest that the observed yields and densities could be sensitive to the boat aggregation and fishing mortality parameter shortly after the MPA implementation, the long-term results seem to be independent of the starting point, and therefore reliable. Nevertheless, in this study we did not included temporal variability, which combined with spatial variability, can have an effect on the optimal MPA size and place. Future simulation should test for a combination of multiple positions and sizes of MPA that can maximize protection and fishery yields.

It is important to highlight that the results in of this work are not a prediction of how a large MPA would work for any particular stock, but are the outcomes of a theoretical exercise to demonstrate that it is possible to use an MPA to recover, protect, and increase yields of an overfished pelagic stock. These results are expected to represent how a large MPA would work for an anchovy like pelagic fish, and would not necessarily hold for other species with different population and fisheries dynamics.

This contribution expands our understanding of how the level of knowledge about fish movement dynamics can affect the design of an effective MPA. The design of large MPAs that successfully protect stocks and increase fisheries yields, even under limited movement dynamics understanding, reinforces the potential for successfully using MPAs as a management tool for pelagic stocks. Furthermore, our methodology and results spur exciting research avenues that can expand the applicability of MPAs; for example, including temporal variability, climate change or economic drivers of the fishing fleet could provide additional realism as well further insight into the value of MPAs for a range of biological and social outcomes.

Through our simulations and comparisons between fish movement scenarios, we systematically assessed the benefit of increasing the complexity of the movement dynamics representation, and explored how that complexity affects the perception of the potential MPA benefits. Our findings demonstrate the value of increasing the understanding about the movement dynamics of the stock, and suggest that large MPAs can be effective as management tool for highly mobile pelagic stocks.
